# Supplementary material for: Tinselenidene: a Two-dimensional Auxetic Material with Ultralow Lattice Thermal Conductivity and Ultrahigh Hole Mobility
Source: Sci Rep. 2016 Feb 1;6:19830. doi: 10.1038/srep19830 (PMC4735295; doi:10.1038/srep19830)
Supplement: Supplementary Information [file srep19830-s1.pdf]

Tinselenidene: a Two-dimensional Auxetic Material with Ultralow Lattice Thermal Conductivity and Ultrahigh Hole Mobility

Li-Chuan Zhang,[†] Guangzhao Qin,[†] Wu-Zhang Fang,[‡] Hui-Juan Cui,[‡] Qing-Rong Zheng,[‡] Qing-Bo Yan,[*,†] Gang Su[*,‡]

†College of Materials Science and Opto-Electronic Technology, University of Chinese Academy of Sciences, Beijing, China 100049,

‡School of Physical Sciences, University of Chinese Academy of Sciences, Beijing, China 100049

Corresponding Author

*Email: (Q.-B.Y.) yan@ucas.ac.cn.

*Email: (G.S.) gsu@ucas.ac.cn.

Contents

1 The geometrical structure of tinselenidene……………………………………………...3

2 The electronic localization function of tinselenidene and phosphorene……...4

3 The binding energy of tinselenidene and other 2D materials……………………..5

4 The geometric parameters and mechanical properties of tinselenidene……..6

5 The band structure of tinselenidene calculated with different methods……..7

6 Projected band structure and density of states of tinselenidene………………...8

7 The geometric phase transition of tinselenidene under strain…………………..9

8 The band gap of tinselenidene modulated by uniaxial stress…………………….10

9 The strain effect on effective carrier mass…..……………………………………….11

10 The effective mass and mobility of carriers…………………………………………..12

11 Young's modulus and effective 2D elastic modulus…………………………….……13

12 The calculation of effective 2D elastic modulus……………………………………..14

13 The calculation of deformation potential constants………………..………………15

14 The calculation of deformation potential constant for CBM C_X…………..……17

15 The technique details in the calculation of lattice thermal conductivity….…..………………………………………………………………………………….…18

16 The fitting details of Possion's ratio in tinselenidene…………………..……….19

References………………………………………………………………………………………….…20

1 The geometrical structure of tinselenidene

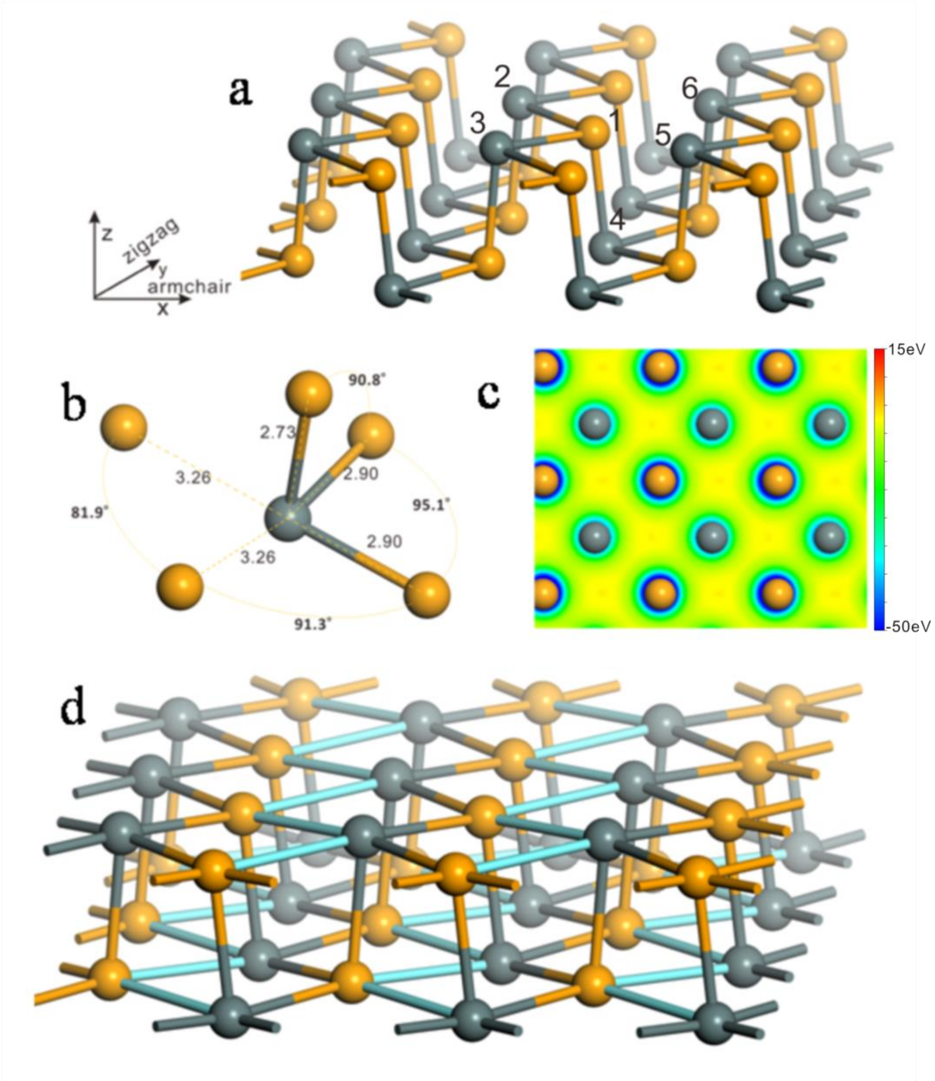

Figure S1 (a) The schematic structure of tinselenidene, (b) a Sn atom surrounded by five neighboring Se atoms, in which the Sn-Se distances and angles are indicated, (c) the electrostatic potential on the plane crossing all Sn atoms, (d) The schematic structure of tinselenidene with all nonbonding neighboring atoms are connected with light blue sticks.

Tinselenidene has a hinge-like structure with a deformed honeycomb structure of graphene, which is anisotropic intuitively as shown in Fig. S1(a). Each Sn (Se) atom is surrounded by five Se (Sn) atoms, three of which are bonded with bond length about 2.90 Å (horizontal bond) and 2.73 Å (vertical bond), while other two are nonbonding neighbor atoms with Sn-Se distance of 3.26 Å, as indicated in Fig. S1(b). The electrostatic potential on the plane crossing all Sn atoms are shown in Fig. S1(c), which is consistent with the electron density in Fig. 1(e), revealing a square-like lattice of tinselenidene. The electrostatic potential around Se atom is obviously deeper than that of Sn atom.

2 The electronic localization function of tinselenidene and phosphorene

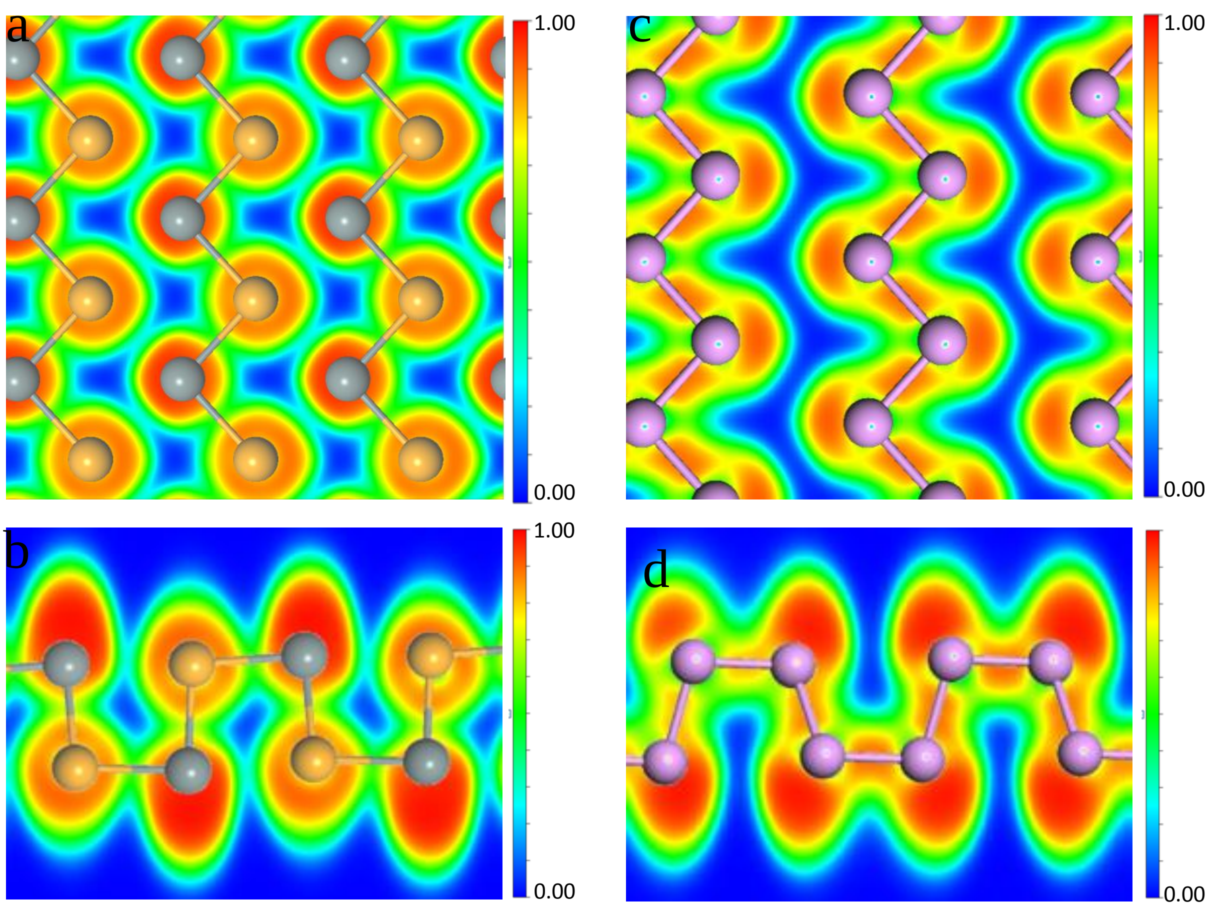

Figure S2 The electronic localization functions of tinselenidene (a, b) and phosphorene (c, d) are illustrated in (001) plane and the plane close to the (110) plane, where yellow, gray and pink balls denote Se, Sn and P atoms, respectively.

3 The binding energy of tinselenidene and other 2D materials

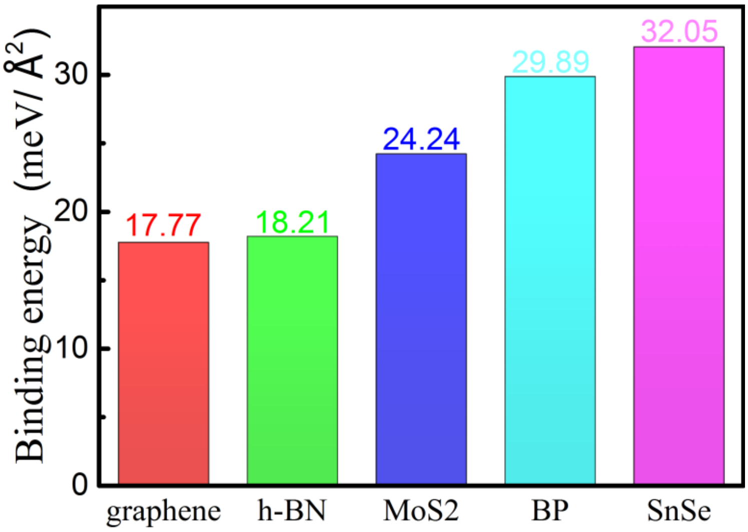

Figure S3 The binding energies of graphene, h-BN, MoS_2, monolayer black phosphorus (phosphorene) and tinselenidene.

The binding energy per unit area indicates the interlayer binding strength of layered solids, which can be defined as follows

$$E_b = \frac{E_{monolayer} - E_{bulk}/2}{S} \quad (1)$$

where $E_{monolayer}$ represents the unitcell energy of tinselenidene, E_{bulk} represents the unitcell energy of bulk SnSe, and S is the unitcell surface area of tinselenidene. Note that a unit cell of bulk SnSe contains two SnSe layers. The binding energy of tinselenidene is nearly double of graphene but close to phosphorene, revealing that the inter-layer interaction of SnSe is stronger than graphene, and tinselenidene may be more difficult to be exfoliated than graphene.

4 The geometric parameters and mechanical properties of tinselenidene

Table S1 Geometrical and mechanical properties of tinselenidene and phosphorene.

	a (Å)	b (Å)	R_{12}=R_{13} (Å)	R_{14} (Å)	R_{15}=R_{16} (Å)	Young's modulus (GPa)		Poisson's ratio (z)		Group velocity (Km/S)	
						x	y	x	y	Γ-X	Γ-X
tinselenidene	4.41	4.27	2.90	2.73	3.26	24.3	43.5	-0.171	0.46	2.90	3.09
Phosphorene[1,2]	4.58	3.32	2.28	2.24	3.41	45.9	165.0	0.046	-0.027	4.01	7.83

The geometrical and mechanical properties of tinselenidene and phosphorene are listed in Table S1 for comparison. Obviously, the geometrical structure of tinselenidene is more symmetric than phosphorene. The Young's module and sound velocity of tinselenidene are much smaller than that of phosphorene, and are less anisotropic. The Poisson's ration occurs between armchair (x) direction and perpendicular z direction for tinselenidene, while that appears between zigzag (y) and perpendicular z directions for phosphorene. Thus the mechanism of negative Poisson's ratio in tinselenidene and phosphorene should be different. The absolute value of negative Poisson's ratio of tinselenidene is 6 times larger than phosphorene.

5 The band structure of tinselenidene calculated with different methods

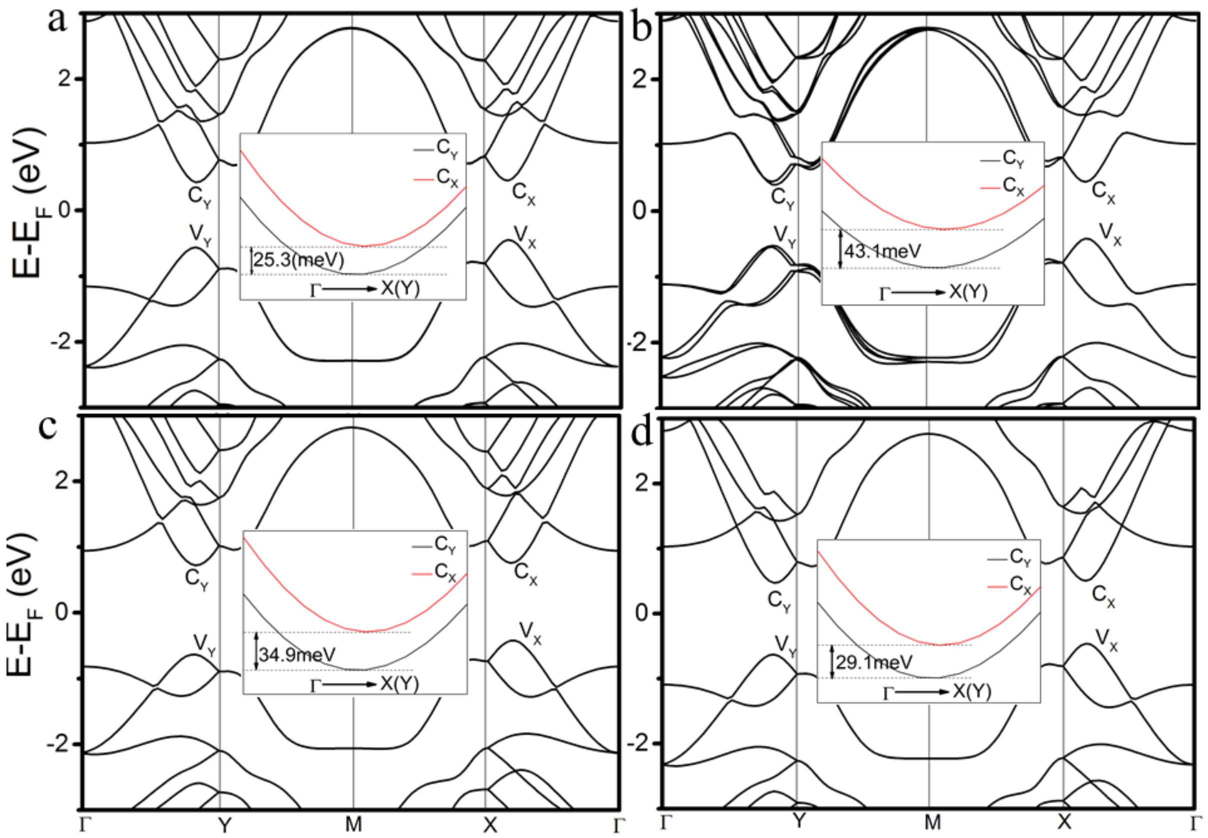

Figure S4 Band Structure of tinselenidene calculated with different methods. (a) Perdew-Burke-Ernzerhof (PBE) functional, (b) PBE functional with spin-orbital coupling has been taken into account, (c) van der Waals interaction has been taken into account using optB88 functional, (d) PBE functional with Sn_d pseudopotential. The zooming of the bands near C_X and C_Y is indicated in the insets by red and black lines, respectively.

Different methods are used to calculate the energy band of tinselenidene. For all of them, the energy at C_Y is smaller than that at C_X, and the energy at V_Y is larger than that at V_X, implying that the band gap of tinselenidene is indeed indirect.

6 Projected band structure and density of states of tinselenidene

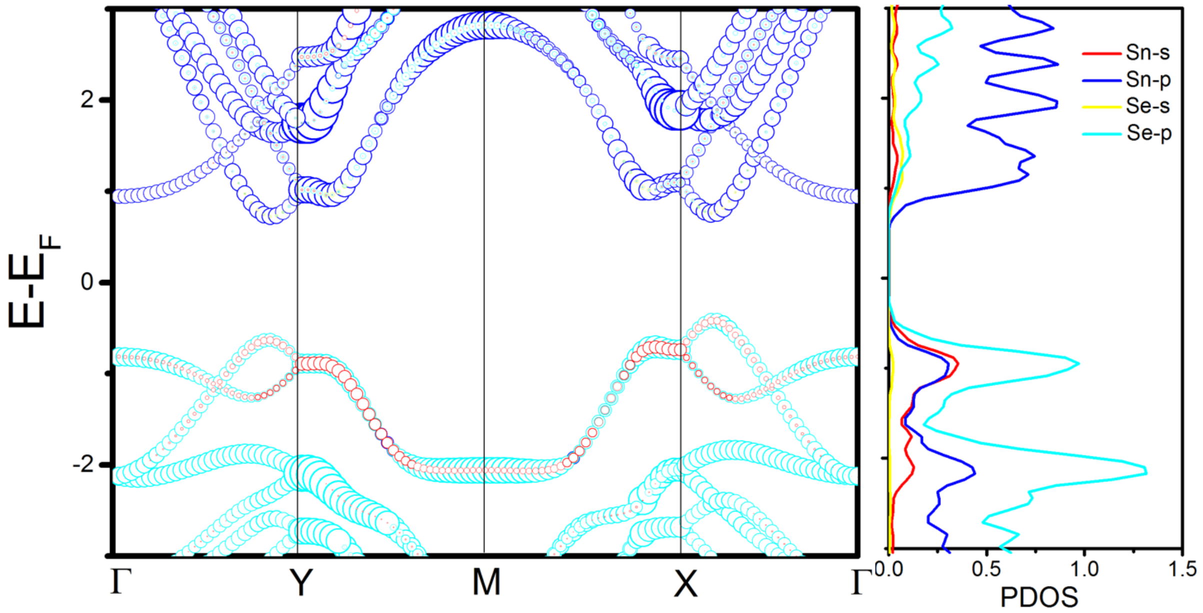

Figure S5 Projected band structure and density of states of tinselenidene. The red, dark blue, yellow and light blue balls (lines) represent the contribution of *s* orbital of Sn, *p* orbital of Sn, *s* orbital of Se, and *p* orbital of Se, respectively.

The calculated projected energy band and density of states are shown in Fig. S5. The major part of valence band and conduction band is derived from the p orbital of Se and p orbital of Sn, respectively, indicating that the electron transfers from Sn to Se, which is consistent with the observation in Fig. 1e that the electron density around Se atom is much higher than that of Sn atom. This charge transfer can be understood when considering the difference in electronegativity between Sn (1.96) and Se (2.55).

7 The geometric phase transition of tinselenidene under a strain

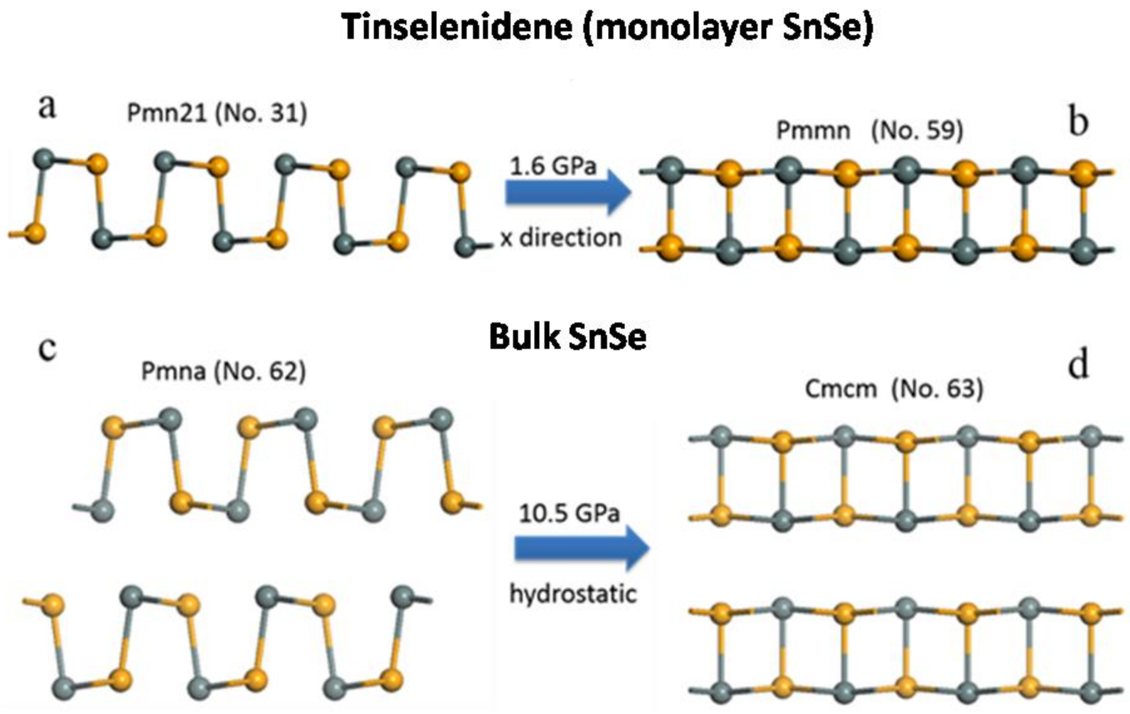

Figure S6 The geometric phase transitions of tinselenidene and bulk SnSe. (a) and (c) indicate the geometrical structure before transition, (b) and (d) indicate the geometrical structure after transition. Note that the unit cell of bulk SnSe contains double layers.

The schematic geometric structures before and after phase transitions of tinselenidene and bulk SnSe [3] are shown in Figure S6, revealing a similar geometrical deformation. Note that as tinselenidene does not have a purely two-dimensional structure, we use the three-dimensional space group to describe its symmetry in a tetragonal supercell.

8 The band gap of tinselenidene modulated by uniaxial stress

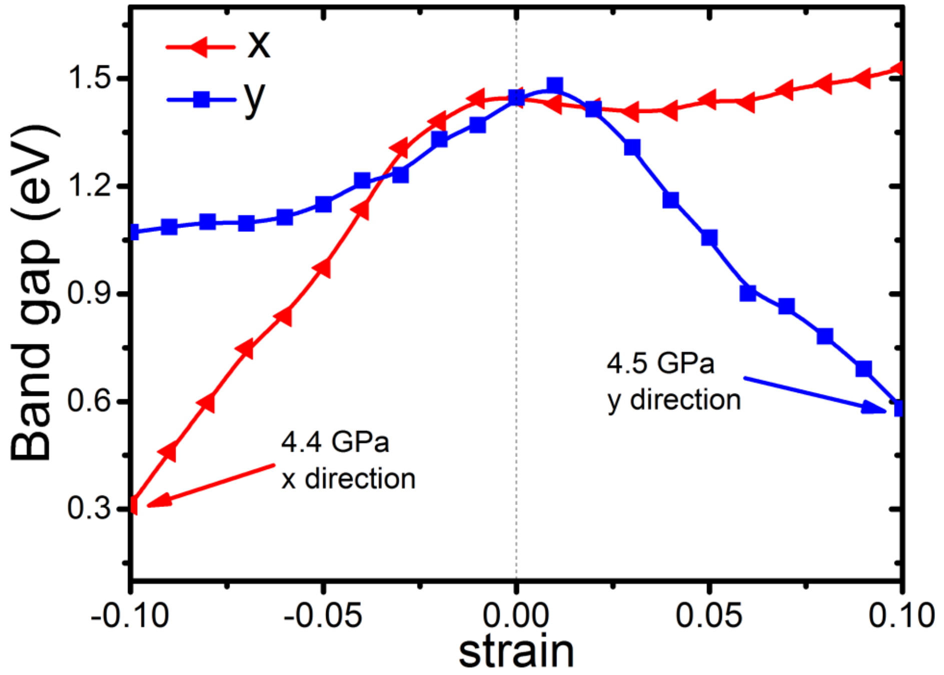

Figure S7 The band gap of tinselenidene under different uniaxial strain. The stress corresponding to 10% compressive strain along *x* direction and 10% tensile strain along *y* direction are indicated.

The bandgap of tinselenidene under different uniaxial strains is shown in Fig. S7. These band gap values can be evaluated from Fig. 4c and 4d. It reveals that a 10% compressive strain along *x* direction and a 10% tensile strain along *y* direction can narrow the energy gap down to 0.3 eV and 0.6 eV, where the stress corresponds to 4.4 GPa and 4.5 GPa, respectively.

9 The strain effect on carrier effective mass

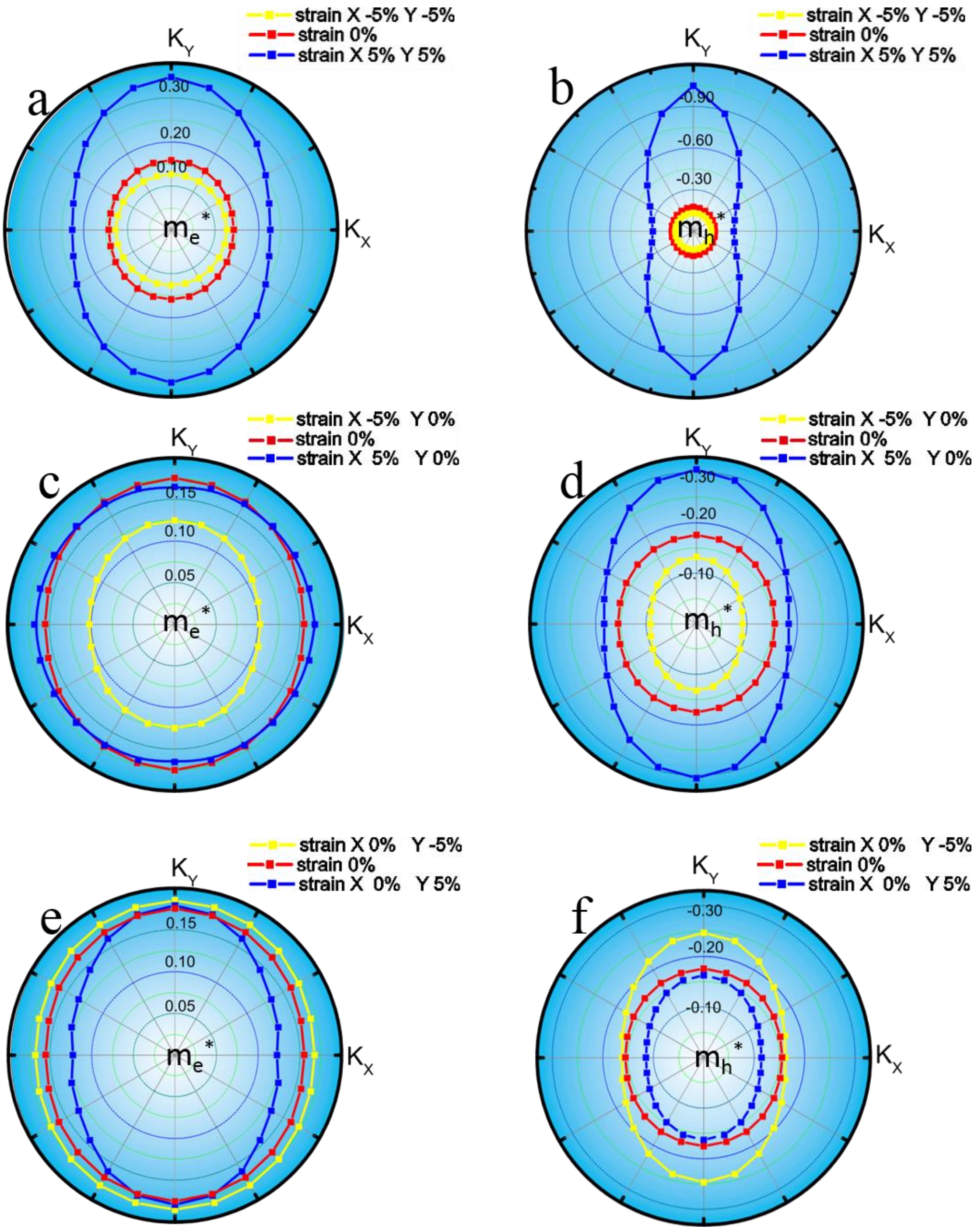

Figure S8 The effective mass of electron (a, c, e) and hole (b, d, f) under different strain.

The effective masses of carrier are shown in Fig. S8. It can be observed that the compressive strain along x direction and the tensile strain along y direction decrease the carrier effective mass, and the biaxial tensile strain dramatically increases the effective mass and induce a noticeable anisotropy.

10 The effective mass and mobility of carriers

Table S2 The effective mass, mobility of charge carriers in tinselenidene.

Carrier type	m_x^*/m_0	m_y^*/m_0	E_{l_x}	E_{l_y}	C_{2D_x}	C_{2D_y}	μ_{2D_x}	μ_{2D_y}
	Γ-X	Γ-Y	(eV)		(Jm^{-2})		($10^3 cm^2V^{-1}S^{-1}$)	
electron	0.143	0.158	-3.28±0.10	4.65±0.13	13.8	25.1	1.20-1.35	0.99-1.10
hole	0.155	0.175	-0.94±0.06	-4.09±0.12	13.8	25.1	11.52-14.88	1.05-1.18
electron (direct, evaluated from C_X)	0.148	0.172	2.73±0.07	-6.21±0.31	13.8	25.1	1.59-1.76	0.46-0.56

Table S2 lists the predicted effective mass and mobility of carriers in tinselenidene. m_x* and m_y* are effective mass in Γ-X and Γ-Y directions, respectively. E_{l_x} (E_{l_y}) and C_{2D_x} (C_{2D_y}) represent the deformation potential constant and effective 2D elastic modulus for *x* (*y*) direction. μ_{2D_x} and μ_{2D_y} denote the carrier mobility in *x* and *y* direction, respectively. Considering that the energy of the conduction band minimum C_X is very close to that of the conduction band bottom C_Y, the carrier properties corresponding to C_X are also calculated and listed in the last row of Table S2 for a comparison.

11 Young's modulus and effective 2D elastic modulus

The Young's modulus reflects the stiffness of elastic materials and can be defined as the ratio of the stress to strain along special axis directions. In the calculation of tinselenidene, the stress value generated by VASP should be rescaled by multiplying a coefficient *z/t* to obtain the real stress, where *z* is the length of cell in z direction and *t* is the effective thickness of tinselenidene (about 5.9 Å, half length of bulk SnSe cell in z direction). The Young's modulus can be evaluated from the slope of stress-strain curve (insets of Fig. 3). Using this method, we calculated the Young's modulus of phosphorene and the result is in good agreement with previous work[4].

In the calculation of carrier mobility, a 2D effective elastic modulus (C_{2D}) is defined, which is similar to the Young's modulus (Y) but is independent of thickness, and can be calculated from the following equation

$$C_{2D} = \frac{1}{S_0}\frac{\partial^2 E}{\partial (l/l_0)^2}\bigg|_{l=l_0} \quad (2)$$

where E and l are the total energy and lattice constant after deformation, l_0 and S_0 are the lattice constant and cell volume at equilibrium for a 2D system.

The relationship between Y and C_{2D} can be expressed as $C_{2D}=Yt$, where t is the effective thickness of tinselenidene. Thus, we have two different methods to calculate Y and C_{2D}, and the results are listed in Table S3. Obviously, they are in good agreement with each other.

Table S3 Young's modulus and effective 2D elastic modulus of tinselenidene.

method	Young's modulus (GPa)		Effective 2D elastic modulus (Jm^{-2} or Nm^{-1})	
	x	y	X	y
Stress-strain	24.3	43.5	14.3	25.6
Differential energy	22.9	42.5	13.8	25.1

12 The calculation of effective 2D elastic modulus

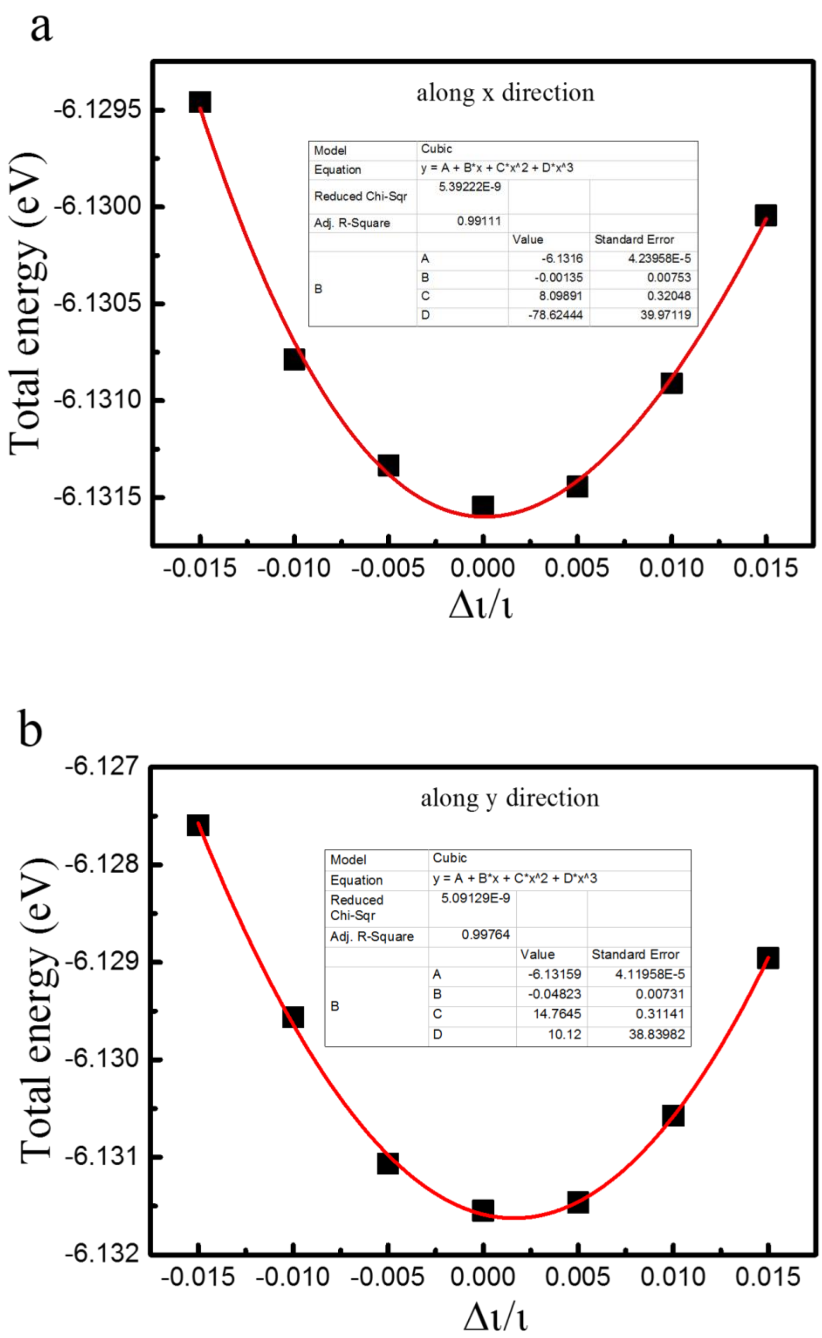

Figure S9 The total energy versus relative change of lattice parameter along x (a) and y (b) directions. Red lines are quadratic fitting curves. From the fitting coefficient, the 2D effective elastic modulus can be derived using Eq. S1.

13 The Calculation of deformation potential constants

a

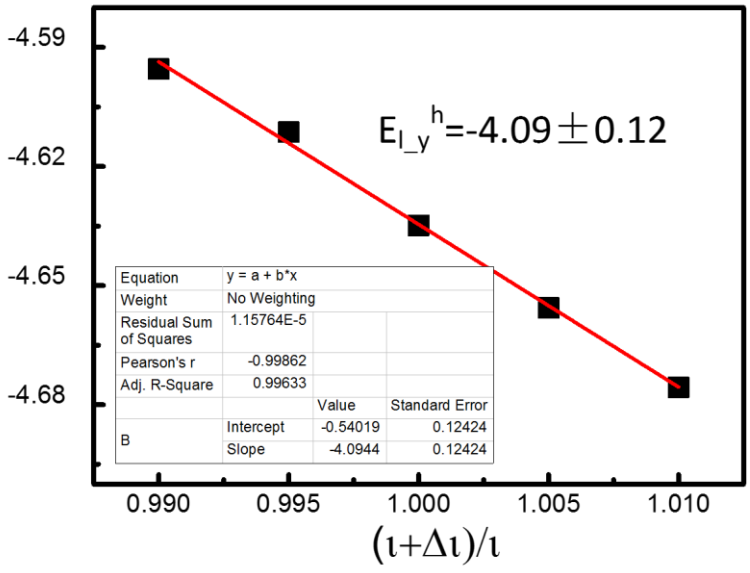

b

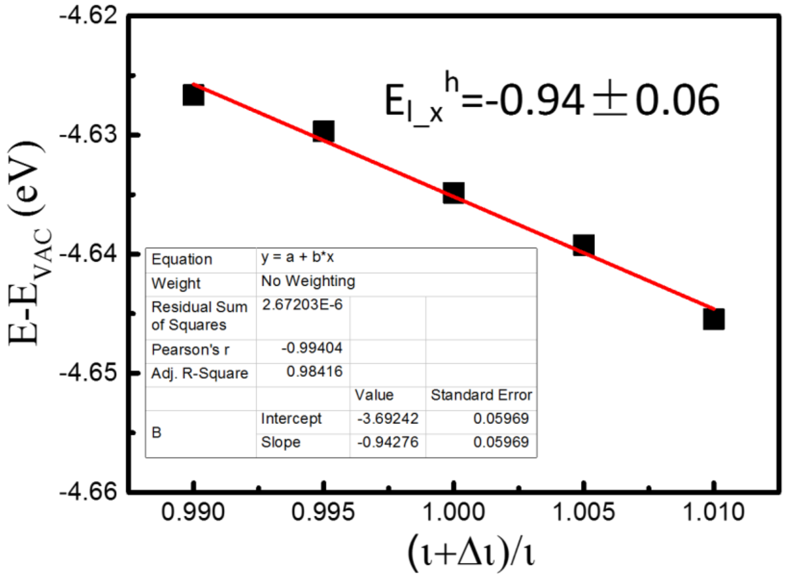

c

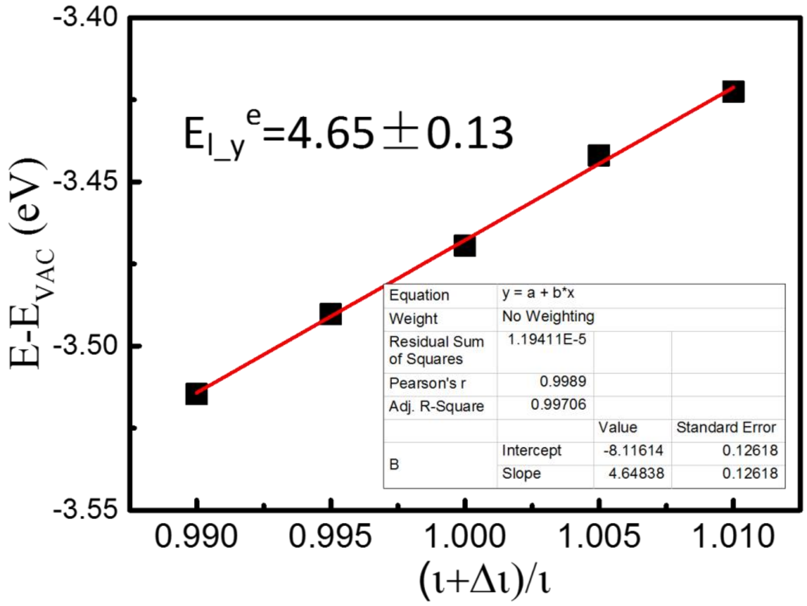

d

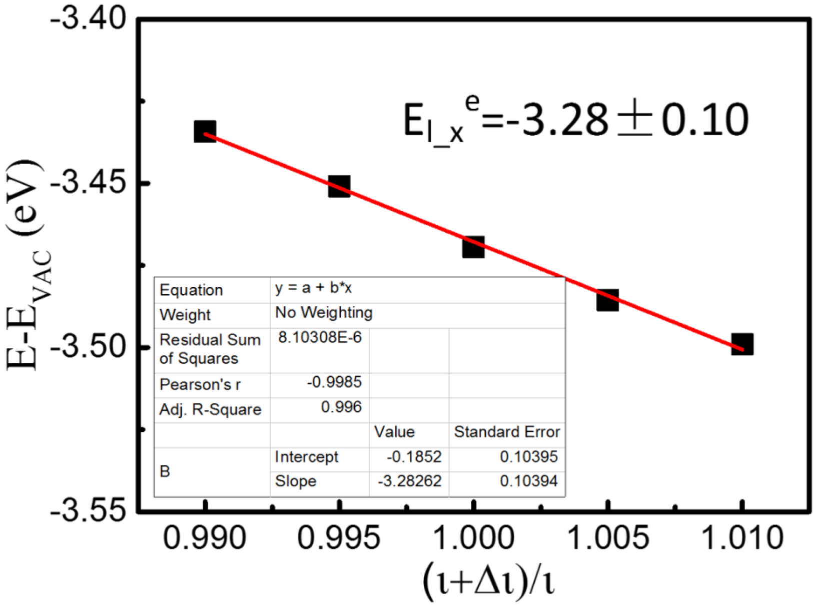

Figure S10 The energies of VBM and CBM versus relative change of lattice parameter along *x* (a, c) and *y* (b, d) directions. Vacuum levels are used as reference energy. Red lines indicate the linear fit, and the slopes of which correspond to deformation potential constants. The standard errors are listed in the insets.

14 The calculation of deformation potential constant for CBM C_Y

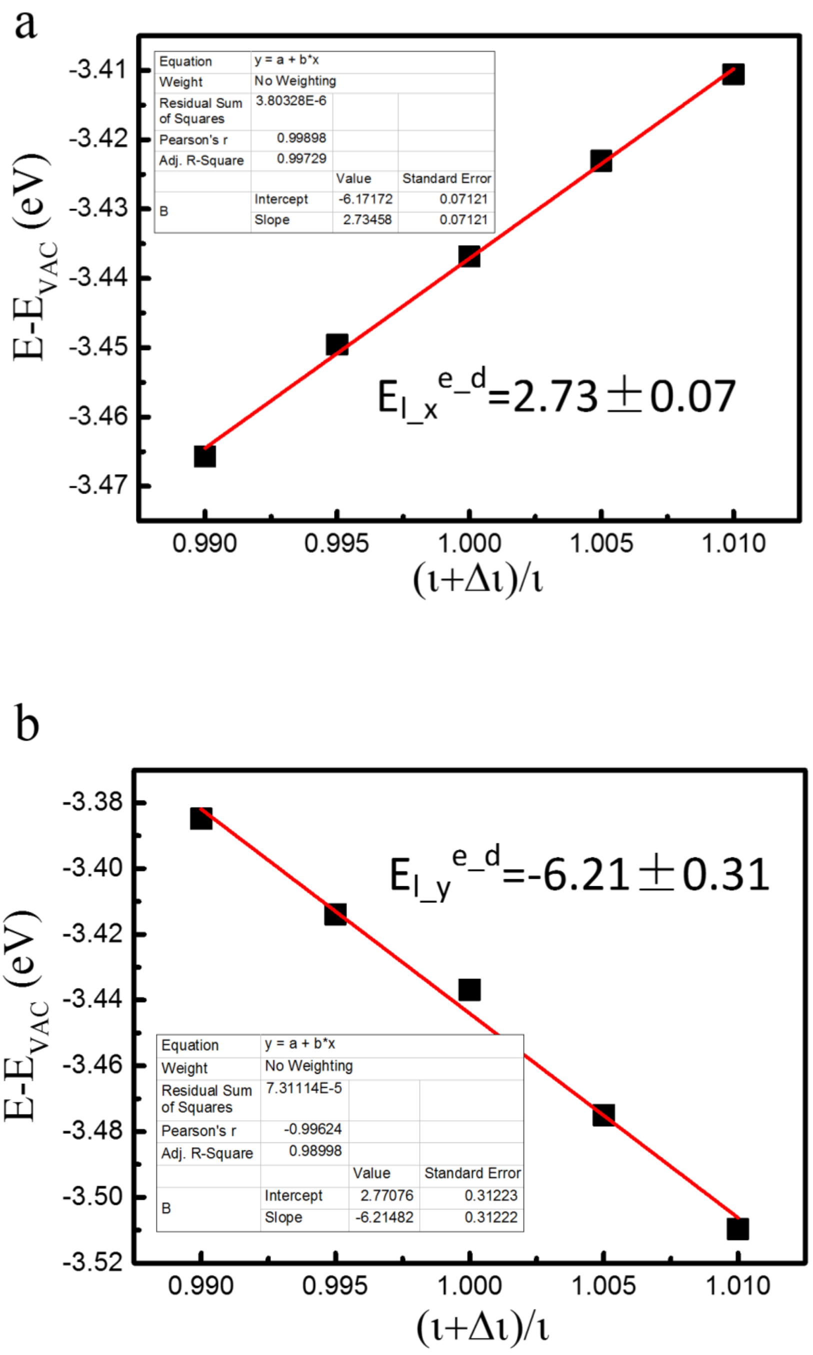

Figure S11 The calculation of deformation potential constant for conduction band minimum C_Y.

Considering that the energy of the conduction band minimum C_Y is very close to that of the conduction band bottom C_X, the deformation potential constant corresponding to C_Y is also calculated as shown in Fig. S11 for a comparison.

15 The technique details in the calculation of the lattice thermal conductivity

When considering only the phonon–phonon scattering processes, the intrinsic lattice thermal conductivity of tinselenidene could be obtained by solving the phonon Boltzmann transport equation as implemented in the ShengBTE code. The finite displacement difference method is employed to get the harmonic and anharmonic third-order interatomic force constants (IFCs), in which $5 \times 5 \times 1$ supercell is used and interactions are taken into account up to fourth nearest neighbors. We additionally obtain the dielectric tensor and the Born effective charges based on DFPT by taking into account long-range electrostatic interactions. As a thickness is necessary in the calculation of thermal conductivity of 2D materials, we choose half the bulk lattice constant along the z direction as the thickness of bulk SnSe, which is about 5.9 Å.

16 The fitting details of Poisson's ratio in tinselenidene

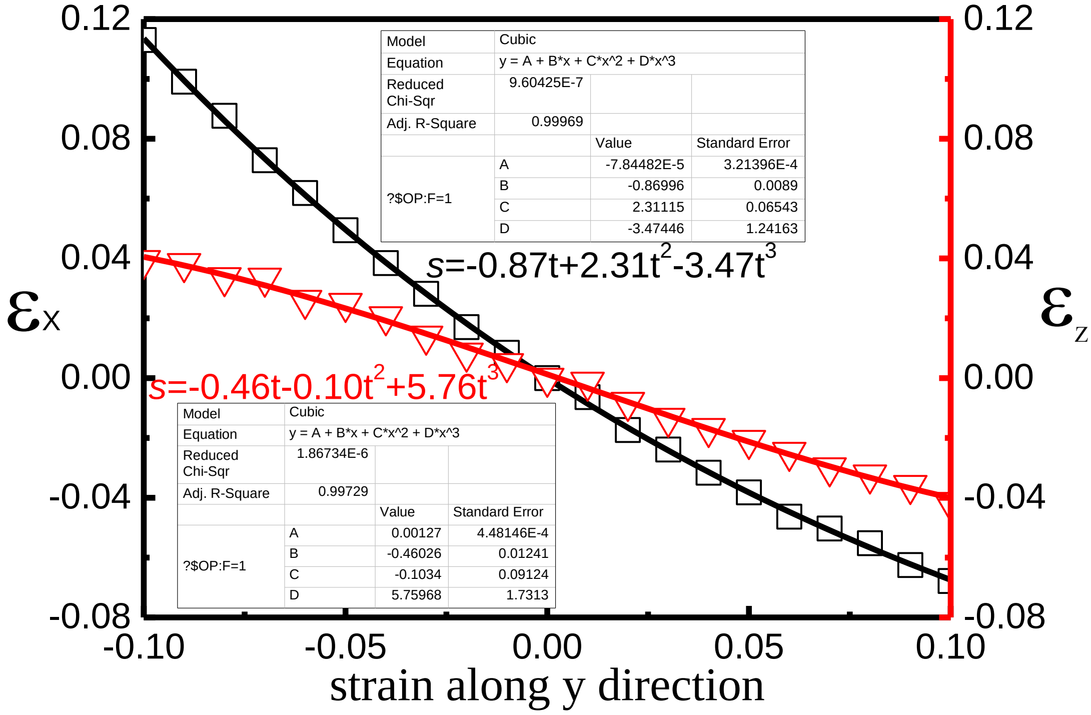

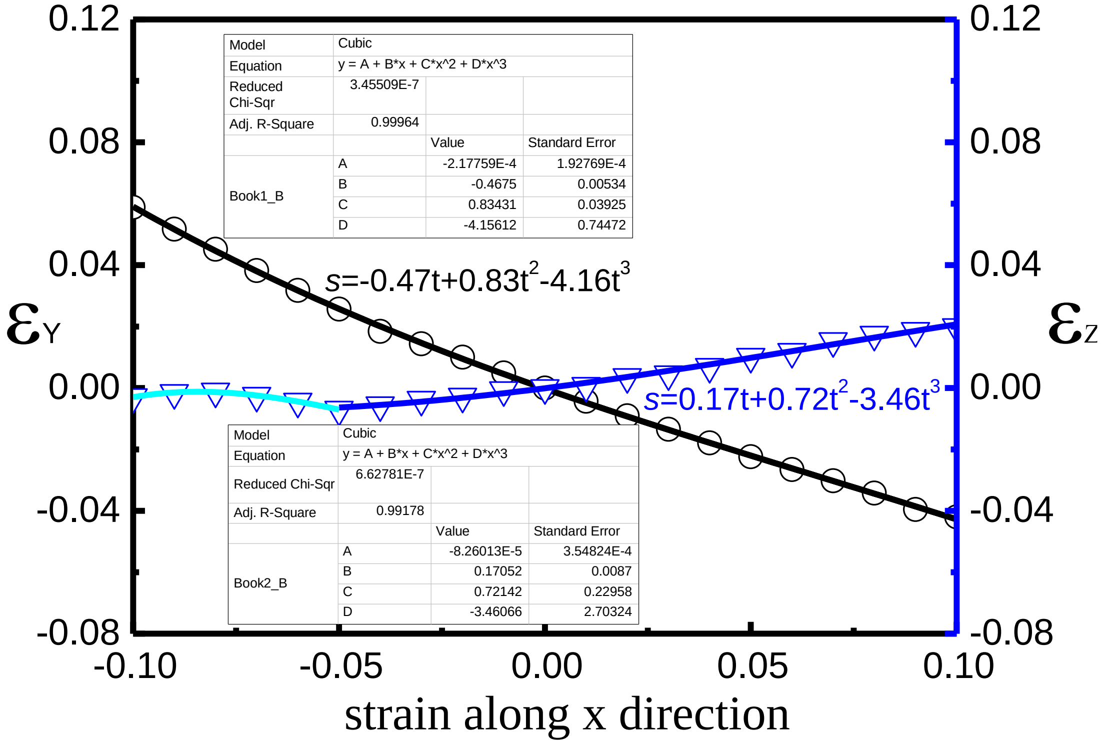

References

[1] Qin, G., Yan, Q.-B., Qin, Z., Yue, S.-Y., Hu, M., Su, G., *Phys. Chem. Chem. Phys. 17*, 4854 – 4858 (2014).
[2] Jiang, J.-W., Park, H. S., *Nat. Commun. 5*, 4727 (2014).
[3] Loa, I. R., Husband, J. R., Downie, A., Popuri, S. R. J.-w. G., *J. Phys.: Condens. Matter 27*, 072202 (2015).
[4] Wei, Q., Peng, X., *Appl. Phys. Lett. 104*, 251915 (2014).
